# Reversal of Immunity After Clearance of Chronic HCV Infection—All Reset?

**DOI:** 10.3389/fimmu.2020.571166

**Published:** 2020-10-08

**Authors:** Heiner Wedemeyer, Tanvi Khera, Benedikt Strunz, Niklas K. Björkström

**Affiliations:** ^1^Department of Gastroenterology, Hepatology and Endocrinology, Hannover Medical School, Hannover, Germany; ^2^Department of Gastroenterology and Hepatology, Essen University Hospital, University of Duisburg-Essen, Essen, Germany; ^3^German Center for Infection Research (DZIF), Hannover-Braunschweig, Germany; ^4^Center for Infectious Medicine, Department of Medicine Huddinge, Karolinska Institutet, Karolinska University Hospital, Stockholm, Sweden

**Keywords:** hepatitis C, chronic infection, direct acting antivirals, soluble inflammatory mediators, natural killer cell, MAIT cell, T cell

## Abstract

Chronic viral infections cause deterioration of our immune system. However, since persistent infections rarely can be eliminated, the reinvigoration capacity of an exhausted immune system has remained largely elusive. Chronic hepatitis C virus (HCV) infection can since some years be effectively cured with novel direct acting antiviral agents. Thus, it is now possible to study reversal of immunity in patients that are cured from a long-lasting chronic infection. We here highlight recent developments in the analysis of various immune cell populations during and after clearance of HCV infection. Surprisingly, whereas reinvigoration of certain immune traits clearly can be seen, many features of immune exhaustion persist over time after viral elimination. Thus, a long-term chronic insult might result in irreversible damage to our immune system. This will be important to consider in therapeutic vaccination efforts against chronic infection and in the development of immunotherapy based strategies against cancer.

## Introduction

Chronic viral infections have a profound impact on the immune system. In humans, it is well established that patients with chronic hepatitis viruses and/or HIV infection have an impaired adaptive immunity with dysfunctional CD4+ and CD8+ T cells contributing to the inability to clear the infection (1, 2). In addition, the natural killer (NK) cell repertoire and function are altered in patients with prolonged viremia from different chronic infections (3–5). Similarly, the mucosal-associated invariant T (MAIT) cell compartment is severely diminished with impaired function in many chronic infections (6), whereas atypical memory B cell accumulates (7). However, the capacity for immune system reinvigoration after elimination of a chronic pathogen remains less well understood. Here, we first review insights related to immune system restoration in chronic infections where virus can be suppressed but not cleared. After that we focus on new results into the possible reversal of immunity after clearance of chronic hepatitis C virus (HCV) infection. This latter research has been possible to perform because of recent paradigm-shifting development in treatment possibilities for chronic hepatitis C, where the vast majority of patients now can clear their chronic infection.

## Evidence for Reversal of Immunity After Suppression, but Not Clearance, of a Chronic Viral Infection in Humans

It has been debated over the years to what extent the profound immune alterations observed in persistent infections could be reversed upon control or elimination of the underlying infection. This has been addressed to some extent in patients with chronic hepatitis B virus (HBV), hepatitis delta (HDV) or HIV infections receiving treatment. HBV replication can be controlled by potent nucleoside or nucleotide analogous (NA) but the infection is not cleared (8). Of note, suppression of viral replication with undetectable HBV viral load did not lead to major functional restoration of HBV-specific T cells (9). HBV-specific immunity only improved in the few patients that managed to clear infection after long-term antiviral therapy (functional cure, HBsAg seroconversion) (10). Although some earlier studies have shown transient restoration of HBV-specific T cells, this short-lived nature of immune restitution represents a favorable condition for virus reactivation (11, 12). Similarly, no full restoration of HIV-specific T cell responses was observed even after the virus had been suppressed for several years with antiviral treatment (13). With respect to NK cells, they are activated and functionally altered in hepatitis B and D virus infection (14, 15). The NK cell phenotype seems to be altered by viral suppression with NA (16), but functional consequences remain unclear. However, the phenotype of NK cells did predict long-term control of hepatitis B after stopping antiviral therapy (17). In chronic HIV infection, the NK cell population is dysregulated in several ways including in their capacity to interact with dendritic cells (18) and with the appearance of dysfunctional CD56^neg^ NK cells (19). However, upon antiviral treatment, some of these alterations are reversed, although it takes two years or longer for them to be normalized (20). Similar to NK cells, also MAIT cells are affected by both chronic HBV and hepatitis D virus infections as well as by HIV with severely reduced numbers in circulation and diminished responsiveness to bacterial challenge or innate cytokine stimulation (21–23). Whereas partial reversal of NK cell immunity was observed upon suppression of HBV, HDV, or HIV infections, no such reversal has been described for MAIT (21–23). However, common for chronic HBV, HDV, and HIV infections is that antiviral treatment only suppresses viral replication and rarely (HBV, HDV) or never (HIV) leads to actual clearance of infection. Thus, although evidence for partial reversal of immunity exists in studies of these infections, the full reinvigoration capacity of the immune system is not possible to gauge since the infections *de facto* are not eliminated.

## The Unique Model of Chronic HCV to Study Reversal of Immunity

In contrast to chronic HBV or HIV, chronic HCV infection can now be efficiently cured by antiviral therapies. Thus, chronic HCV infection represents a unique model to study host–pathogen interaction in humans and to investigate the effects of clearance of a persistent long-term infection on the immune system. As a background, infection with HCV becomes chronic in 50–90% of adults where it manifests as chronic liver disease including development of cirrhosis, liver failure, and hepatocellular carcinoma (24). Treatment of hepatitis C virus underwent fundamental changes in late 2013. Before then, antiviral therapy was based on administration of pegylated interferon alfa in combination with ribavirin curing approximately half of the patients but with severe side effects (25–27). In November 2013, the first interferon-free treatment option was approved for the treatment of chronic hepatitis C. Since then many additional direct acting antivirals (DAAs) became available. These DAAs are either targeting the HCV protease, the HCV NS5A protein which is involved in HCV replication and packaging of virions, or the HCV polymerase. Importantly, these regimens have basically no side effects and response rates have been shown to exceed 97%, not only in clinical trials, but also in real world treatment settings, and successful treatment leads to regression of clinical symptoms and complications of liver disease (28, 29).

Thus, with this remarkable development, it is now possible, for the first time, to study immune system function in well-controlled large cohorts of patients that become cured from a chronic infection. This is of both basic immunological and clinical relevance. In more detail, new basic knowledge on the inherent capacity of immune system reinvigorated after a prolonged chronic insult will be important for exposure to other heterologous pathogens, development of immune mediated diseases, immune control of malignancies, and also for vaccine design. Furthermore, and in the HCV context, the previously infected patients may become re-exposed to HCV, and it is currently unclear if those patients need to be re-treated or if they have a chance to spontaneously control HCV due to restored antiviral immunity. Indeed, successfully treated chronic hepatitis C patients still have a risk to develop hepatocellular carcinoma (30). In this setting, HCV clearance may interfere with immune surveillance of malignant cells, and thus a better insight into the effects of rapid HCV removal on innate and adaptive immunity is of interest.

In the sections below, we discuss recent insights that have been gained in the last couple of years with respect to immune restoration following removal of chronic hepatitis C. In addition, we summarized various recent studies on immune cells and their fate after HCV clearance in **Table 1**.

**Table 1:** Summary of different immune cell populations and their fate after antiviral therapy.

	Authors	Type of patient	No. of patients	Treatment(follow up-EOT)Weeks	Main alterations observed upon HCV clearance
**Soluble immune mediators**					
	Carlin et al. (31)	CHC (cirrhotic and non- cirrhotic)	131	SOF/RBV (16 or 20)	Four inflammatory markers were measured, IP-10, MCP-1, MIP-1*β*, and IL-18, and all decline during therapy but display different dynamics after therapy. MIP-1*β* and IP-10 displayed significant difference based on treatment outcome.
	Hengst et al. (32)	CHC (cirrhotic & NASH)	53	SOF/RBV (36)	IP-10, IL-12 p40, IFN alpha 2a, IL-18, and TRAIL were upregulated in comparison to NASH and healthy controls. The changes in SIM were not fully reversible upon clearance of viral infection.
	Debes et al. (33)	CHC & NASH	13	SOF/NS5A/PI (+/−RBV) 96	Normalization of innate immunity after viral clearance.
	Gorin et al. (34)	CHC & AC	28	SOF+PI+3D+NS5A/NA(+/−RBV) (36 or 48)	Rapid restoration of plasma cytokine milieu observed. Macrophage activation marker s CD163 remained elevated. In addition elevated levels for CSCL10 and sCD14 were observed whereas CCL5 and IL-4 remained suppressed.
**T cells**					
**CD8**	Martin et al. (35)	CHC	51	NA+PI +/−RBV (24)	Specific phenotypic changes were observed on CD8 T cell but expression of CD127 and PD-1 on global CD8 T cells were not altered by therapy. Specific restoration of CD8 T cell proliferation.
	Weiland et al. (36)	CHC	24	NS5A SOF/3D +/− RBV (8–12)	HCV-specific CD8T cells were analyzed that displayed T cell exhaustion and memory like characteristic both before and after therapy. Only CD127+/PD1+ subset maintained after clearance. The subset characterized by high expression of transcription factor TCF1.
	Aregay et al. (37)	CHC	40	SOF/PI/3D/NS5A +/−RBV (24)	Surface expression of co-regulatory receptors on exhausted HCV-specific CD8 T cells remained unaltered. Mitochondrial dysfunction of exhausted HCV-specific CD8 T cells was not restored. HCV-specific CD8 T cells remained functionally impaired after clearance.
**CD4**	Smits et al. (38)	CHC	248	SOF/PI/3D/NS5A +/−RBV (24)	HCV-specific CD4 T cells skewed towards a follicular T helper cell phenotype maintained after clearance.
**Tregs**	Langhans et al. (39)	CHC	14	SOF+PI/NS5A (54)	Increased frequency and activation status of Tregs that do not normalize even after long term follow-up.
**γδ T cells**	Ravens et al. (40)	CHC & non cirrhotic	23	SOF+NS5A (48)	NGS and flow cytometeric sorting was performed to monitor the peripheral γδ TCR repertoire and their clonal distribution. Overall clonality and complexity of TCR γδ was comparable to healthy. The γδ T cell compartment and their associated TCR repertoires were highly stable at a long term follow up.
	Ghosh et al. (41)	CHC	24	SOF+NS5A/PI (12)	Peripheral Vγ9Vδ2 γδ T cells showed phenotypic and functional alterations despite cure. CD38 expression in SVR group was not different from healthy but declined at the EOT but relapsers had higher CD38+ frequencies.
**MAIT cells**	Hengst et al. (42)	CHC	26	SOF+ RBV (72)	MAIT cells present in lower frequencies, circulating MAITs display altered phenotype, impaired in MR1 dependent function. Function and cell frequency not restored after elimination of virus, no correlation with clinical parameters and liver disease.
	Spaan et al. (43)	CHC	22	PI/NS5A (24) SOF/NS5A+/−RBV (24)	MAIT cell frequencies decreased in all cohorts. No association between the frequency of MAIT cells and ALT level, HCV RNA, and liver fibrosis score. Patients with differential fibrosis stage showed similar MAIT frequencies.
	Bolte et al. (44)	CHC	42	SOF+NS5A (24)	Paired liver biopsies and blood samples were studied. MAIT cell frequency was lower in blood and liver compared to healthy. Liver MAIT cells displayed higher activation and cytotoxicity than MAITs from blood. Impaired MR1 dependent cell function.
	Cannizzo et al. (45)	HCV/HIV co-infected, IFN non responders, cART treated	5	NS5A/PI/SOF/3D +/− RBV (24)	At baseline diminished total CD3 and CD8 MAITs compared to healthy and no recovery. Longitudinally. MAIT subsets showed higher CD69, PD-1, and granzyme expression but no difference in CD39 and Il-18R and perforin expression.
**Natural killer (NK)**					
	Serti et al. (46)	CHC	13	NS5A/PI (24)	Post therapy decrease in NK cell activation and a normalization of NK cell cytotoxic effector functions to healthy. Paired liver biopsies showed similar normalization trend.
	Spaan et al. (47)	CHC	12	NS5A/PI (12)	NK cell frequencies altered to levels comparable to healthy. NK cell functions (IFNγ and perforin) not modulated.
	Strunz et al. (48)	CHC	35	SOF/+ RBV (96)	NK cells from patients with chronic HCV maintained their functional capacity. Chronic infection reduced NK cell diversity and this reduction persisted long after viral clearance.
	Wang et al. (49)	CHC	26	SOF/NS5A (24 or 36)	Significant decline in CD56^bright^ NK cell frequencies that normalize after EOT but no difference in CD56^dim^ NK cells observed. Expression levels of NKG2A, NKp30, and CD94 were high at baseline but recovered to levels those of healthy after EOT.
	Jiang et al. (50)	CHC	13	SOF/NS5A (24 or 36)	Expression of functional markers were downregulated after treatment but the potential activity of NK cells gets upregulated. Amongst the NK markers, NKp46 normalized at EOT.
	Golden-Mason et al. (51)	Prospective cohort	22	LDV/SOF (24)	Transient activation followed by dampening of NK cell activity to pre- treatment levels
	Mele et al. (52)	CHC	59	DAA (24)	Restoration of normal adaptive NK phenotype (activation markers normalized) and restored ADCC ability.

CHC, chronic hepatitis C; NASH, non-alcoholic steatohepatitis; AC, alcoholic cirrhotic; SOF, sofosbovir; RBV, ribavirin; PI, Protease inhibitor; NA, nucleot(s)ide analogs; 3D-Ombitasvir/Paritaprevir/Ritonavir + Dasabuvir; EOT, end of treatment; NK, natural killer cells; MAIT, mucosal associated invariant T cells; TCF1, transcription factor; cART, combination antiretroviral therapy.

## HCV Clearance and Effect on Systemic Pro-Inflammation

**Figure 1:**
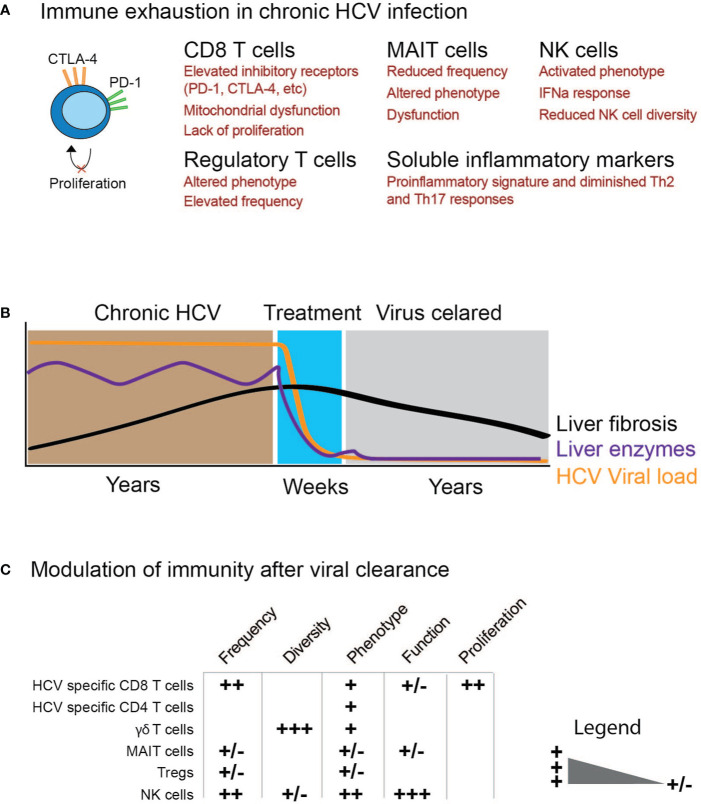
Schematic illustration of immune cell function before and after elimination of chronic HCV infection. **(A)** Examples of possible immune exhaustion as a consequence of chronic HCV infection. **(B)** Time course for studying the impact of HCV clearance on immune cells. **(C)** Modulation of immunity after viral clearance with the degree of change ranging from (+/−) to (+++).

Chronic HCV causes a distinct inflammatory milieu by inducing IFN stimulated genes (ISGs) which impacts clinical manifestations of HCV infection and even tumor development. Upon chronic HCV infection, hepatocytes are triggered to produce type I and III IFNs which induce production of ISGs with antiviral activity (5). Despite this induction of the interferon system, the majority of patients establish chronic infection. In patients with chronic hepatitis C, a variety of systemic pro-inflammatory cytokines and chemokines are elevated (32, 34). In addition, the systemic inflammatory repertoire is different in HCV-infected patients compared to patients with non-alcoholic steatohepatitis (NASH) a non-viral chronic disease (32). This elevation affects different pro-inflammatory cytokines, chemokines, and growth factors including CXCL10 (IP-10), IL-12, IFN-α, IL-18, and TRAIL (32, 34). An obvious question is if these changes are driven by HCV infection or just secondary to underlying liver inflammation and/or liver disease. Indeed, elevated levels of VCAM-1 and ICAM-1 have been shown to be associated with the degree of liver fibrosis (32). In long-term follow-up studies after clearance of infection with DAA treatment, many of the pro-inflammatory cytokines and chemokines returned to normal levels albeit a residual signature with elevated levels of IFN-α and TRAIL persisted months after viral clearance (32, 34). Overall, persistent HCV infection is associated with profound alterations in levels of soluble inflammatory mediators which are related with liver disease progression, treatment outcome and viral presence. Importantly, these changes were not fully reversible upon viral clearance (**Table 1**, **Figure 1**).

## Partial Reinvigoration of CD4+ and CD8+ T Cells Upon Viral Clearance

Initial studies investigating HCV-specific T cells in patients receiving DAA therapies suggested a partial restoration of effector functions, in particular of antigen-specific T cell proliferation (35). Still, the level of restoration was heterogeneous, and not all patients normalized T cell function. These findings were supported by other studies showing that suppression of HCV replication led to a decline in T cell exhaustion marker expression and an increase in HCV-specific IFN*γ* responses after treatment (53–55). However, HCV-specific CD8+ T cells with phenotypic features of exhaustion and memory potential can survive in an antigen-independent manner, both during and after DAA therapy and HCV clearance (36). This survival might be mediated by expression of the transcription factor TCF-1 (36). However, a restoration of HCV-specific CD8+ T cell exhausted surface phenotype does not, *per se*, translate to full functional reinvigoration. Indeed, our group recently reported that the mitochondrial and metabolic dysfunction of virus-specific CD8+ T cells persisted despite viral clearance (37). However, other data suggests that mitochondrial function may partially improve in some patients (but not in all patients) during antiviral therapy (56).

With respect to CD4+ T cells, antiviral treatment of HCV led to a shift from a Th1 to a follicular helper T cell (Tfh) signature within HCV-specific cells (38). Similar to HCV-specific CD8+ T cells, Tfh cells are likely persisting in an antigen-independent manner (38). Moreover, regulatory T cells are usually found in higher numbers in chronic hepatitis C and, surprisingly, these increased Treg frequencies with an activated phenotype persisted also during and after DAA therapy of HCV infection (39) (**Table 1**).

In addition, it has been recently shown that the transcription factor TOX is crucial during appearance and maintenance of exhausted T cells in mice (57–59). Of note, in humans, it is clearly shown that HCV-specific CD8T cells remain TOX positive after DAA mediated elimination indicating a chronic scar (59). Whereas TOX was suggested to be a master regulator of exhausted T cells in mice, other recent work in humans has shown that, except for being expressed on exhausted T cells, also effector memory T cells express this transcription factor (60). Finally, the effect of HCV therapy on T cells specific to other antigens such as CMV and EBV (61) as well as on T cells recognizing tumor antigens (62) has been studied. Here, molecules indicating activation of T cells decreased in expression levels over time, but no major functional changes were observed in the majority of cases. **Table 1** summarizes the effect of DAAs on T cells.

Overall, studies investigating T cell responses suggest that viral clearance and lack of ongoing antigen stimulation lead to a down-regulation of T cell activation and exhaustion markers but antigen-specific dysfunction is not restored—even when patients are followed for up to a year after HCV elimination. Antigen-independent survival of distinct subsets of virus-specific CD4+ and CD8+ T cells has been reported, and these populations constitute potential targets for immunotherapy to prevent HCV re-infection (36). Long lived T cell memory is often observed during spontaneous resolution of acute hepatitis C infection both in humans and chimpanzees. These memory CD4 and CD8 T cells appear important for rapid control of secondary infection. In a recent study the data suggested that CD8T cell memory was rather narrow after successful treatment with DAA, and the authors suggested that vaccination maybe one option to induce the broader memory response which may provide protective immunity (63).

## Unconventional T Cells in Clearance of Chronic HCV

Compared to conventional CD4 or CD8 T cells, unconventional T cells, such as *γδ* T cells and MAIT cells are typically rapid effector cells that respond within hours towards foreign antigens and/or other innate signals exhibiting important functions during viral infection (6, 64, 65). In chronic hepatitis C, *γδ* T cells are less efficient in producing cytokines and exhibit an activated phenotype (41, 66). Whereas the activated phenotype vanished upon DAA-mediated HCV clearance, the dysfunction remained (41). This dysfunction was not due to a skewing in the T cell receptor repertoire as it was shown to be unaltered in patients with chronic hepatitis C and further remained stable after elimination of HCV (40). This is distinct compared to chronic HIV infection where the repertoire is heavily altered because of the infection but slowly returns to normal after prolonged antiviral treatment (67).

Compared to *γδ* T cells, MAIT cells have been more extensively studied in the context of chronic HCV infection. MAIT cells are highly enriched in the human liver and they efficiently respond to innate cytokines such as IL-12, IL-18, and IFN-α suggesting that they exhibit an important role in the antiviral immunity against HCV (68, 69). However, in chronic hepatitis C, MAIT cell numbers are reduced both in liver and peripheral blood (42–45). In fact, of all alterations in peripheral blood immune cell subsets in chronic hepatitis C, loss of MAIT cells was shown to be the major phenotype (42). Loss of MAIT cells in chronic hepatitis C appears to both be a consequence of the infection, *per se*, but also to the underlying liver disease as patients with liver cirrhosis tend to have reduced numbers of MAIT cells (70, 71). Loss of MAIT cells was accompanied with MAIT cell activation with increased expression of CD69, HLA-DR, PD-1, and granzyme B (42). Despite having an activated phenotype, MAIT cell function, in response to bacterial challenge but not innate cytokine stimulation, was hampered in chronic hepatitis C (42, 44) (**Table 1**). This phenotype of MAIT cells observed in chronic hepatitis C is similar to what has been described for infections with HBV, HDV, and HIV (21–23). Upon treatment and viral clearance, circulating MAIT cell numbers remain suppressed for years (42) whereas a certain restoration of intrahepatic MAIT cells following viral clearance have been noted (44). However, MAIT cell activation and dysfunction remained (42, 44), albeit with some reversal of the activated signature noted in one study (44). The inability for MAIT cells to become reinvigorated upon pathogen removal appears to be shared among chronic infections as similar findings have been reported for chronic HBV, HDV, and HIV infections (21–23). The long-term consequences of having this “gap” in the immune system are currently unknown. However, it is interesting to note that patients with chronic viral hepatitis infections progressing to end-stage liver disease have an increased risk for bacterial infections (72). This might partly be due to a dysfunctional MAIT cell compartment (71). Future research should focus on identifying the signals needed for restoring the MAIT cell compartment. Some insight into this came from a recent study showing that *in vivo* IL-7 administration significantly expanded the human MAIT cell compartment (73).

## Impact of Chronic HCV and Clearance Thereof on NK Cells

Similar to MAIT cells, also NK cells are highly enriched in the human liver (74) and thus have been extensively studied in the context of chronic HCV infection (5, 75). Both genetic and cellular studies have revealed an important role for NK cells in the control of HCV infection (5, 75–77). However, in chronic hepatitis C, NK cell phenotype and function are compromised at multiple levels (15, 78, 79). Upon antiviral treatment and rapid clearance of HCV, several groups have in recent years assessed whether the compromised NK cell compartment recovers (**Table 1** and **Figure 1**). Interestingly, when measuring single parameters of NK cell “health”, both phenotype and function seem to partly, or fully, normalize upon viral clearance. This includes reversal of an aberrant phenotype with normalized expression of activation and inhibitory receptors such as NKp30, NKp46, TRAIL, and NKG2A (46, 47, 49, 50, 80). This reversal in NK cell phenotype happened within months after viral clearance and was also associated with restored NK cell function (46, 49, 50, 81). The signal responsible for this restoration currently remains unknown. Future work should determine whether this is an active reinvigoration *via* certain signaling pathways or rather the removal of the virus and possibly the ensuing resolution of inflammation that leads to a seemingly restored NK cell compartment.

Diversity is an essential feature of our immune system. While this term has been mostly associated with adaptive immune responses, recent work has also shown that NK cells represent a highly diverse population of immune cells (82, 83). A recent study performed a high-dimensional analysis of NK cells in chronic HCV and treatment thereof (48). It revealed that chronic HCV infection increased inter-individual, but decreased intra-individual, NK cell diversity. This occurred independent of underlying CMV infection, a potent influencer of NK cell repertoire formation and NK cell diversity (84, 85) but could partly be linked to the degree of underlying liver disease (48). Intriguingly, the altered NK cell diversity appeared irreversible since it persisted for at least two years after clearance of chronic HCV. Thus, distinct from single measurements of NK cell function, that appears to normalize upon clearance of a chronic pathogen (46, 47, 49, 50, 51, 80) global affection on the NK cell compartment still remain for years (52). The impact of altered NK cell diversity on an individual’s immunological health in the longer perspective should now be the focus in future studies.

## Important Unanswered Questions

Despite a plethora of recent studies investigating the capacity of the immune system to reset after removal of chronic hepatitis C, several important questions remain. More detailed studies on exhausted HCV-specific CD4+ and CD8+ T cells in chronic HCV are warranted since there might be a degree of heterogeneity with subpopulations of exhausted HCV-specific cells becoming fully reinvigorated after DAA-mediated clearance of the virus. Additionally, various other environmental and host factors may influence the evolution of HCV-specific T cells before, during, and after antiviral therapy including stage of liver disease, sex, and age. These factors may also explain differences between different cohorts. Furthermore, several additional arms of the immune system still remain to be studied in the context of DAA treatment of hepatitis C patients including myeloid immune cells and HCV-specific B cells. Out of necessity, most of the above described work have focused on immune cells in peripheral blood. However, researchers should in the future also strive towards finding means to access and interrogate the intrahepatic immune environment in relation to rapid clearance of chronic HCV (44, 86). Additionally, studies on the epigenetic imprint of immune cells after successful treatment are also warranted. The growing understanding of epigenetic gene regulation as it relates to both the stability and malleability of T cell memory may offer the potential to selectively modify T cell memory in disease by targeting epigenetic mechanisms (87). Underlying this are alterations at the chromatin level that regulate constitutive and inducible gene expression including histone modification and DNA methylation (88). Some studies have demonstrated that HCV infection modifies the position of histone modifications, thereby inducing an epigenetic signature that persists following the cure with DAAs and these changes can be reverted by specific drugs. This may further provide an opportunity for prevention of HCC progression (87, 89). It is well accepted that HCV cure does not eliminate the short term risk to develop hepatocellular carcinoma. Moreover, there has been concern that HCC recurrence rates may even be higher in patients who had received curative first line therapies for HCC and who subsequently received DAA therapy against chronic hepatitis C. In a recent paper from our group we showed that HCC surveillance may indeed be affected by DAA therapy of chronic HCV infection and identified that IL-12 could be a key player in the regulation of HCC-specific CD8+T cell responses (62). Finally, although some of the published studies have longitudinally characterized patients for up to almost 2.5 years after viral clearance (48), with the large number of chronic hepatitis C patients now be cured, longitudinal studies aiming at following patient cohorts for at least 5, or even 10 years, are now feasible and will provide an even better estimate of our immune systems inherent capacity to recover.

## Conclusions

In this brief review, we highlight recent development in the analysis of various immune cell populations during and after clearance of chronic HCV infection. This represents the first human model where a pathogen successfully can be eliminated after years of chronic infection allowing us to determine long-term consequences of immunity following resolution of this insult. Although there has been evidence on antigen-independent survival mechanisms, including a role for TCF-1, of HCV-specific T cells which could represent targets for immune interventions. However, surprisingly many imprints of chronic HCV infection on distinct immune compartments persist for years despite antigen elimination.

## Author Contributions

TK and BS drafted the figure and table and wrote parts of the review. HW and NB drafted the layout for the review and wrote the discussion. All authors contributed to the article and approved the submitted version.

## Funding

This work was funded by the by the Deutsche Forschungsgemeinschaft (DFG, German Research Foundation)—Projektnummer 158989968—SFB 900, SFB738 of the German Research Foundation (DFG), German Centre for Infection Research (DZIF), internal funds from the university of Duisburg-Essen, Swedish Research Council, the Swedish Cancer Society, the Swedish Foundation for Strategic Research, Knut and Alice Wallenberg Foundation, the Novo Nordisk Foundation, the Center for Innovative Medicine at Karolinska Institutet, the Stockholm County Council, Karolinska Institutet.

## Conflict of Interest

HW reports grants and personal fees from Abbvie, grants, personal fees, and non-financial support from Abbott, grants, personal fees, and non-financial support from Roche Diagnostics, personal fees from Siemens, grants and personal fees from BMS, grants and personal fees from Gilead, grants and personal fees from Novartis, grants and personal fees from Roche, personal fees from Janssen, grants and personal fees from Merck/MSD, grants and personal fees from Eiger, grants and personal fees from Falk and Falk Foundation, other from Transgene, non-financial support and other from Myr-GmbH, all outside the submitted work; and HW received honoraria for consulting and research support by companies developing diagnostic tools and antiviral therapies for hepatitis B and C.

The remaining authors declare that the research was conducted in the absence of any commercial or financial relationships that could be construed as a potential conflict of interest.

## Abbreviations

CMV, cytomegalovirus; DAA, direct acting antiviral; EBV, Epstein bar virus; ICAM-1, intercellular cell adhesion molecule-1; *γδ*T cells, gamma-delta T cells; MAIT, mucosal-associated invariant T cells; NA, nucleos(t)ide analog; NK cells, natural killer cells; VCAM-1, vascular cell adhesion molecule.
